# Dynamic Changes in the Human Milk Metabolome Over 25 Weeks of Lactation

**DOI:** 10.3389/fnut.2022.917659

**Published:** 2022-07-14

**Authors:** Katrine Overgaard Poulsen, Fanyu Meng, Elisa Lanfranchi, Jette Feveile Young, Catherine Stanton, C. Anthony Ryan, Alan L. Kelly, Ulrik Kraemer Sundekilde

**Affiliations:** ^1^Department of Food Science, Aarhus University, Aarhus, Denmark; ^2^Sino-Danish Center for Education and Research, Aarhus, Denmark; ^3^School of Food and Nutritional Sciences, University College Cork, Cork, Ireland; ^4^ACIB – Austrian Centre of Industrial Biotechnology, Graz, Austria; ^5^Teagasc Food Research Centre, Moorepark, Cork, Ireland; ^6^Brookfield School of Medicine and Health, University College Cork, Cork, Ireland

**Keywords:** human milk, metabolomics, term delivery, lactation, NMR spectroscopy

## Abstract

Human milk (HM) provides essential nutrition for ensuring optimal infant growth and development postpartum. Metabolomics offers insight into the dynamic composition of HM. Studies have reported the impact of lactation stage, maternal genotype, and gestational age on HM metabolome. However, the majority of the studies have considered changes within the first month of lactation or sampled with large intervals. This leaves a gap in the knowledge of progressing variation in HM composition beyond the first month of lactation. The objective of this study was to investigate whether the HM metabolome from mothers with term deliveries varies beyond 1 month of lactation, during the period in which HM is considered fully mature. Human milk samples (*n* = 101) from 59 mothers were collected at weeks 1–2, 3–5, 7–9, and 20–25 postpartum and analyzed using ^1^H nuclear magnetic resonance spectroscopy. Several metabolites varied over lactation and exhibited dynamic changes between multiple time points. Higher levels of HM oligosaccharides, cis-aconitate, O-phosphocholine, O-acetylcarnitine, gluconate, and citric acid were observed in early lactation, whereas later in lactation, levels of lactose, 3-fucosyllactose, glutamine, glutamate, and short- and medium-chain fatty acids were increased. Notably, we demonstrate that the HM metabolome is dynamic during the period of maturity.

## Introduction

Feeding a full-term infant with mother’s milk provides macronutrients and non-nutritional compounds accommodating the infant’s needs in the early neonatal period following birth, including supporting growth, immunological development, and gut maturation (1, 2). Human milk (HM) contains immunological compounds interacting with the developing infant’s immune system (3), non-nutritional compounds involved in immunological and growth-related metabolic mechanisms (2, 4), and vitamins and minerals (5), as well as prebiotic glycans, such as human milk oligosaccharides (HMOs), providing energy for the development of the gut microbiota and ensures antimicrobial activity in the gut (6, 7). Thus, the composition of HM is complex and is in addition dynamic over time, with intraindividual variation observed for various types of constituents (8–12).

Human milk metabolomics is still a developing scientific field with continual changes and improvements in analytical technology. One of the most used techniques is NMR spectroscopy as this method is robust, quantifies the metabolites in high concentration, and yields a good coverage of milk metabolites across different metabolite classes (13). Recently, metabolomic studies have contributed to insight into low-molecular weight compounds of HM and their variation (8, 14). Studies have particularly reported variations in mono- and disaccharides, HMOs, free amino acids, free fatty acids, lipids, and intermediates of energy metabolism in HM (15–17), and some of these constituents have implications on infant development. For instance, HMOs are the prebiotic components important for immune system development and gut microbial colonization (6), glutamine and glutamate act as neurotransmitters for the brain besides from serving as nitrogen sources (18), and choline is important in brain development and phospholipid synthesis among others (19).

Studies on HM metabolome provide an opportunity for understanding the impact of maternal genotype, disease, and lifestyle on HM composition reflected by the metabolome (8, 14). The composition of metabolites in HM varies over lactation, with increasing levels of lactose and short- and medium-chain fatty acids and decreasing levels of HMOs when progressing from colostrum (first day after birth) to mature milk (recognized as 4 to 6 weeks postpartum) (2, 15). Apart from the variation during the lactation period, metabolomic studies have demonstrated variation in HM metabolome dependent on maternal genotype, impacting the chemical composition of HMOs in the milk (20). More than 150 different structures dependent on glycosidic linkages and the composition of carbohydrate monomers have been characterized (21). In particular, secretor status and Lewis gene type of the mother influence the chemical structures of HMOs available in HM (12). Mothers classified as secretors excrete HMOs with α1-2 fucosylation, of which 2-fucosyllactose (FL) and lacto-N-difucohexaose (LNDFH) I are among the quantitatively most dominant, whereas the milk of non-secretors lacks these structures. In contrast, non-secretor mothers secrete milk dominated by 3-FL, lacto-N-fucopentaose (LNFP) II and lacto-N-tetraose (LNT) (22). Finally, Lewis negative mothers cannot secrete milk with α1-4 fucosylation and thus lack HMOs such as LNDFH I and II (12). Moreover, variation in HM from mothers diagnosed with preeclampsia (23), dependent on geographical origin (24, 25) and maternal obesity (26–28) has been observed.

Mothers are encouraged to exclusively breastfeed their infant for up to 6 months (29), but we know very less of the metabolic composition of HM over the course of prolonged lactation. The aims of this study were to identify and quantify metabolites in HM from term deliveries and elucidate the impact of lactation stage on HM metabolome from term delivery over 6 months of lactation at various time points. Here, we show that the changes in levels of particularly HMOs occur during the entire lactation period, with levels of 6-siallylactose (SL), LNT, and LNDFH I progressively declining at all lactation stages, and 3-FL gradually increasing. Moreover, we show that most changes are observed within the first month of lactation, but levels of gluconate, glutamine, and O-acetylcarnitine significantly vary between all lactation stages investigated, and levels of other metabolites, including 2-aminobutyrate, choline, ethanolamine, and O-phosphocholine, changed significantly after 1 or 2 months of lactation.

## Materials and Methods

### Recruitment of Participants

The results presented in this study are a part of a larger cohort – INFAMILK, established in Cork, Ireland. The cohort seeks to characterize HM from mothers delivering birth at term over 25 weeks of lactation and further associate HM composition to infant gut microbial development. The subjects providing HM for this study were recruited from Cork University Maternity Hospital, Cork, Ireland, in the time period 2016–2020. Ethical approval for this study was granted by the Clinical Research Ethics Committee of the Cork Teaching Hospitals, Cork, Ireland. All participants enrolled in this study were healthy, provided written consent, and all relevant guidelines and regulations were followed.

Information on infant weight at birth, birth mode, gestational age (GA) at birth, infant sex, mother’s pre-pregnancy body mass index (BMI), gravidae, and parity was recorded. All mothers included breastfed their infants exclusively.

### Sample Collection

This sub-study comprises HM samples from 59 term deliveries. In total, 101 HM samples were collected from the 59 mothers at four different time points postpartum; weeks 1–2 (*n* = 27), 3–5 (*n* = 28), 7–9 (*n* = 31), and 20–25 (*n* = 15) postpartum.

Fresh human milk (10–20 ml) was expressed from mothers in a sterile container, stored at 4°C, and delivered to the laboratory within 24 h. Milk sub-samples were then split and frozen at −80°C immediately and shipped on dry ice to Aarhus University, Department of Food Science, where they were immediately placed in −80°C freezers until analysis.

### ^1^H Nuclear Magnetic Resonance Spectroscopy Metabolomic Analysis of Human Milk

The 101 HM samples intended for ^1^H nuclear magnetic resonance (NMR)-based metabolomics were processed in a random order as followed using standard protocol for milk-based metabolomics (17). Samples were thawed in water bath and kept on ice while Amplicon Ultra 0.5-ml 10-kDa spin filters (Millipore, Billerica, MA, United States) were being washed three times. The samples were skimmed by centrifugation at 4,000 g, at 4°C for 10 min, fat layer removed, and 500 μl of the skimmed milk transferred to individual Amplicon Ultra 0.5-ml 10 kDa spin filters. Next, the skimmed milk was filtered by centrifugation at 10,000 g at 4°C, for 30 min and 400 μl of filtered milk from each sample was transferred to an individual 5-mm NMR tube. In each NMR tube, 200 μl D_2_O with 0.05% 3-(trimethylsilyl)propanoic acid (TSP, Sigma-Aldrich, Saint-Louis, MO, United States) was added. Spectra acquisition was acquired according to the study of Sundekilde et al. (17). Using a Bruker Avance III 600 spectrometer equipped with a 5-mm ^1^H TXI probe (Bruker BioSpin, Rheinstetten, Germany), ^1^H NMR spectra were acquired at 298 K and a ^1^H frequency of 600.13 MHz. A single 90° pulse experiment (Bruker pulse sequence: noesypr1d) was run to acquire one-dimensional spectra with a relaxation delay of 5 s. During the relaxation delay, water suppression was performed, and a total of 64 scans were comprised of 32.768 data points with a spectral width of 12.15 parts per million (ppm). The resulting ^1^H NMR spectra were all referenced to TSP signal at 0 ppm. A line-broadening function by 0.3 Hz was applied to each ^1^H NMR spectra, following by a Fourier transformation. Preprocessing of ^1^H NMR spectra was subsequently conducted by phase and baseline corrections, done both automatically and manually using Topspin 3.2 (Bruker Biospin, Rheinstetten, Germany).

### Identification of Metabolites and Statistical Analyses

Metabolites of the processed ^1^H NMR spectra were identified using Chenomx NMR suite 9.0 (Chenomx Inc., Edmonton, AB, Canada) with Chenomx standard metabolite library and an in-house metabolite library.

Multivariate data analyses of the quantified metabolites were conducted using SIMCA 16 (MKS Data Analytics Solutions, Umea, Sweden). The dataset comprising the quantified metabolites was UV-scaled prior to the analysis. A principal component analysis (PCA) model was computed to evaluate overall variation in the dataset. Potential outliers were identified by inspecting the Hotellings T^2^ plot with a cutoff point at 99%, and residuals plot DModX provided by SIMCA.

Univariate statistical analyses of quantified metabolites were conducted using R statistical environment^1^ (version 4.0.3). Inter-group differences comprised grouping according to the week postpartum. If quantified metabolites adhered to normal distribution, analysis of variance (ANOVA) was conducted and inter-group differences analyzed *post hoc* by Tukey’s honestly significant difference (HSD) test with a level of significance at *p* < 0.05. If quantified metabolites did not adhere to the normal distribution, inter-group differences were analyzed by the Kruskal–Wallis test following the Wilcoxon rank sum test with Benjamini–Hochberg false discovery rate corrected *p*-values with a level of significance at *p* < 0.05.

## Results

### Subject Characteristics

The cohort consisted of 59 mothers delivering birth at term, of whom 30 provided samples at one time point throughout the study period, 18 provided two HM samples, nine provided three and two provided samples at all four time points (weeks 1–2, 3–5, 7–9, and 20–25, Table 1). As further presented in Table 1, the GA of the mothers was on average of 39.6 weeks, and they had an average BMI of 25.95 kg/m^2^ and had given birth once before; 66% underwent spontaneous vaginal delivery, and 25.4% had Caesarean section, in line with population data from publicly-funded deliveries (30). The female/male infants’ ratio was quite even, including about 47.5% females, and all infants were within the normal weight range at birth (2,500–4,500 g) (Table 1).

**TABLE 1:** Subject characteristics of cohort with continuous data presented as means, ± standard deviation and number of observations in round brackets.

Descriptive and clinical information	Cohort (59)
Mothers collecting samples at one/two/three/four different time-points (n)	30/18/9/2
GA at birth (weeks)	39.60 ± 0.98 (58)
Pre-pregnancy BMI (kg/m^2^)	25.95 ± 4.95 (50)
BMI groups (NW/OW/OB) (n)	25/19/6 (50)
Delivery mode (C-section/SVD/Other) (n)	15/39/5 (59)
Gravida	2 ± 1.42 (59)
Parity	2 ± 0.93 (59)
Infant birth weight (g)	3674 ± 449 (58)
Infant sex (F/M)	28/31 (59)

*Categorical data are presented as number of observations included in each category. Abbreviations: BMI; body mass index, C-section; Caesarean section, F; female, GA; gestational age, M; male, n; number of individuals, NW; normal weight, OB; obese, OW; overweight, SVD; spontaneous vaginal delivery.*

### Lactation Stage Drives Overall Variation in the Human Milk Metabolome

We identified 54 different metabolites, including 19 amino acids and derivatives, 11 metabolites related to energy metabolism, 9 fatty acids and derivatives, and 12 sugar metabolites across the 101 samples analyzed. As secretor status of the mother influences the chemical structures of HMOs available in HM (20), mothers were identified as non-secretors or secretors dependent on the presence of the predominant resonance in 2-FL, an α1-2 fucosyl linkage (resonance signal at δ5.32 ppm). In total, 19% of mothers were classified as non-secretors. Further, Lewis status was identified dependent on the presence of an α1-4 fucosyl linkage in LNDFH I and II (resonance signal at δ5.03 ppm). Of the secretor mothers, six were identified as Lewis negative, corresponding to 10% of the total cohorts. We conducted a multivariate data analysis to analyze the overall variation in the metabolome of term HM by computing a PCA model. One of the samples had high levels of several amino acids (leucine, isoleucine, methionine, valine, phenylalanine, tyrosine, Supplementary Figures 1A,B) and, as this observation interfered with variation explained in PCA, the sample was excluded in the following analyses. The final model was summarized in six principal components (PCs) and described 57.4% of the variance in the data.

**FIGURE 1:**
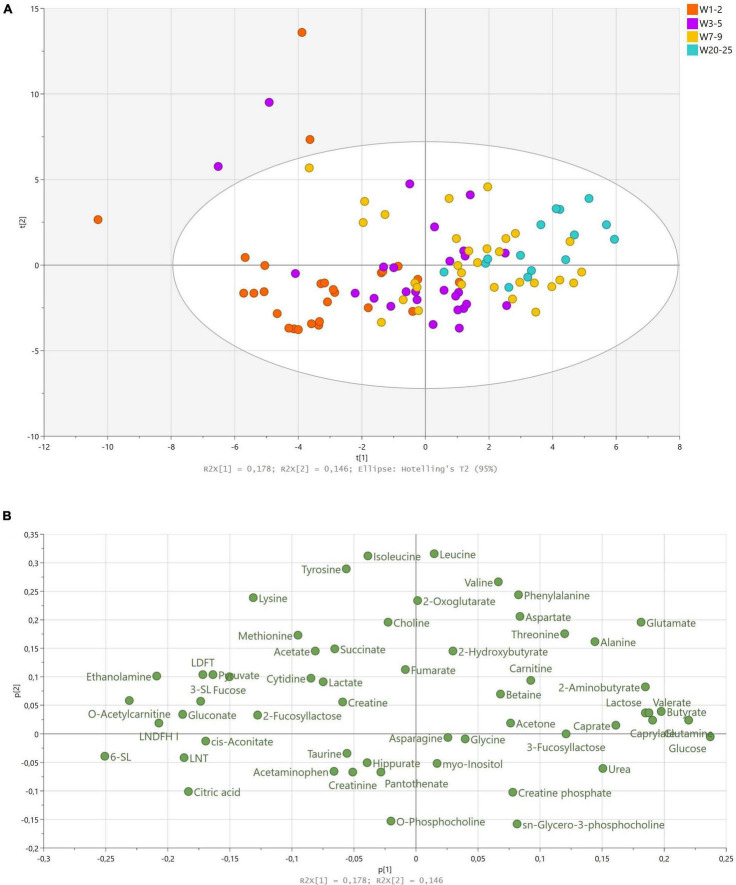
Principal component analysis of human milk samples (*n* = 100) from term deliveries collected from weeks 1 to 25 postpartum. **(A)** Scores scatter plot of observations collected colored according to sampling time postpartum. The legend denotes color-coded time points from weeks 1–2 to 20–25. **(B)** Corresponding loadings scatter plot of variables measured. Abbreviations: LDFT; lactodifucotetraose, LNDFH; lacto-N-difucohexaose, LNT; lacto-N-tetraose, SL; sialyllactose, W; week

The first and second PC accounted for 32.4% of the total variation in the dataset and largely reflected variation over lactation from weeks 1–2 to 20–25. Samples of earlier stages of lactation clustered at the left side of the first PC, whereas samples of later stages of lactation clustered on the right side of the first PC, accounting for 17.8% of the variation in the data (Figure 1A). Samples from weeks 3–5 and 7–9 largely overlapped across the left and right sides of the first PC, indicating that discrimination between weeks 3–5 and 7–9 was not possible using the first two PCs (Figure 1A). The second PC mainly described some samples with high levels of lysine, leucine, isoleucine, and tyrosine as compared to the remaining samples (Figure 1B). We found no influence of delivery mode, maternal pre-pregnancy BMI, gravida, parity, infant birth weight, or infant sex on the metabolome. Maternal secretor status largely separated the samples at the fifth PC, corresponding to approximately 6% of the total variation in the dataset (Supplementary Figure 2A).

As lactation stage was the largest source of variation in the data, we analyzed the corresponding loadings plot accordingly (Figure 1B). The vast majority of HMOs identified (2-FL, 3-SL, 6-SL, LNT, LNDFH I, lactodifucotetraose; LDFT) correlated with samples from earlier stages of lactation (weeks 1–2 and 3–5), whereas 3-FL correlated with later stages of lactation (Figure 1B). Moreover, citric acid, cis-aconitate, gluconate, ethanolamine, O-acetylcarnitine, pyruvate, and fucose correlated with samples from weeks 1–2, and lactose, caprate, caprylate, butyrate, glutamine, glutamate, glucose, 2-aminobutyrate, alanine, urea, and threonine correlated with samples from weeks 7–9 and 20–25 (Figure 1B).

### Concentrations of Metabolites in Human Milk Change Across Multiple Lactation Stages

Coherently, to quantify the statistical importance of the relations observed in the multivariate data analysis, we analyzed the significant differences in the levels of metabolites between lactation stages by univariate analyses (Tables 2–5).

**TABLE 2:** Mean levels (in mM ± standard deviation) of sugars and human milk oligosaccharides in human milk from term deliveries sampled weeks 1–2, 3–5, 7–9, and 20–25 postpartum.

*Sugars and HMOs*	Week 1–2 (mM ± SD)	Week 3–5 (mM ± SD)	Week 7–9 (mM ± SD)	Week 20–25 (mM ± SD)
**2-FL** *	2.48^a^ ± 0.76	1.83^b^ ± 0.78	1.76^b^ ± 0.63	1.31^b^ ± 0.43
**3-FL**	0.94^a^ ± 0.83	1.57^b^ ± 1.04	2.15^bc^ ± 1.88	2.61^c^ ± 1.13
**3-SL**	0.18^a^ ± 0.05	0.15^b^ ± 0.05	0.12^c^ ± 0.04	0.12^bc^ ± 0.03
**6-SL** *	0.95*^a^* ± 0.25	0.55^b^ ± 0.18	0.33^c^ ± 0.14	0.08^d^ ± 0.04
Fucose	0.22 ± 0.15	0.16 ± 0.16	0.16 ± 0.11	0.20 ± 0.08
**Gluconate**	0.90^a^ ± 0.50	0.60^b^ ± 0.29	0.44^c^ ± 0.30	0.25^d^ ± 0.21
**Glucose** *	0.94^a^ ± 0.39	1.33^b^ ± 0.43	1.49^b^ ± 0.52	1.70^b^ ± 0.40
**Lactose** *	166.72^a^ ± 16.08	180.98^b^ ± 15.74	188.56^bc^ ± 15.41	200.76^c^ ± 9.95
**LDFT**	0.68^a^ ± 0.75	0.53^ab^ ± 0.61	0.41^b^ ± 0.43	0.38^ab^ ± 0.14
**LNDFH** I	1.19^a^ ± 0.43	0.95^b^ ± 0.33	0.70^c^ ± 0.29	0.40^d^ ± 0.19
**LNT**	3.82^a^ ± 1.26	2.75^b^ ± 1.07	1.96^c^ ± 0.83	1.13^d^ ± 0.54
Myo-inositol	0.78 ± 0.21	0.74 ± 0.24	0.82 ± 0.26	0.66 ± 0.24

Significant differences were analyzed by one-way ANOVA followed by Tukey’s HSD with a level of significance at p < 0.05. If variables were not normally distributed, statistical significance between time points was analyzed by the Kruskal–Wallis test following the Wilcoxon rank sum test with Benjamini–Hochberg corrected p-values and a level of significance at p < 0.05. Significant differences between time points are indicated by different letters. Metabolites that significantly differ over lactation are written in bold. Abbreviations: FL; fucosyllactose, HMOs; human milk oligosaccharides, LDFT; lactodifucotetraose, LNDFH; lacto-N-difucohexaose, LNT; lacto-N-tetraose, mM; millimolar, SD; standard deviation, SL; sialyllactose, *variables normally distributed.

**TABLE 3:** Mean levels (in mM ± standard deviation) of amino acids and their derivatives in human milk from term deliveries sampled weeks 1–2, 3–5, 7–9, and 20–25 postpartum.

*Amino acids and derivatives*	Week 1–2 (mM ± SD)	Week 3–5 (mM ± SD)	Week 7–9 (mM ± SD)	Week 20–25 (mM ± SD)
**2-aminobutyrate**	0.01^a^ ± 0.00	0.01^a^ ± 0.01	0.01^b^ ± 0.01	0.02^c^ ± 0.01
**Alanine**	0.18^a^ ± 0.12	0.25^b^ ± 0.09	0.24^b^ ± 0.08	0.26^b^ ± 0.05
Aspartate	0.05 ± 0.04	0.06 ± 0.03	0.06 ± 0.02	0.08 ± 0.07
**Betaine**	0.06^a^ ± 0.01	0.07^ab^ ± 0.02	0.07^ab^ ± 0.01	0.08^b^ ± 0.01
Carnitine	0.03 ± 0.01	0.04 ± 0.01	0.04 ± 0.01	0.04 ± 0.03
**Glutamate** *	0.89^a^ ± 0.57	1.25^b^ ± 0.43	1.45^b^ ± 0.48	1.51^b^ ± 0.32
**Glutamine**	0.07^a^ ± 0.05	0.23^b^ ± 0.13	0.41^c^ ± 0.24	0.59^d^ ± 0.23
Glycine*	0.58 ± 0.12	0.58 ± 0.08	0.61 ± 0.14	0.65 ± 0.15
Isoleucine	0.01 ± 0.01	0.01 ± 0.01	0.01 ± 0.01	0.01 ± 0.00
**Leucine**	0.02^a^ ± 0.02	0.03^ab^ ± 0.02	0.03^b^ ± 0.01	0.03^b^ ± 0.01
**Lysine**	0.04^a^ ± 0.05	0.02^b^ ± 0.01	0.02^b^ ± 0.01	0.02^b^ ± 0.01
**Methionine**	0.01^a^ ± 0.01	0.01^b^ ± 0.00	0.01^b^ ± 0.00	0.01^b^ ± 0.00
**O-acetylcarnitine**	0.04^a^ ± 0.02	0.02^b^ ± 0.02	0.01^c^ ± 0.01	0.01^d^ ± 0.00
**Phenylalanine**	0.01^a^ ± 0.01	0.01^ab^ ± 0.01	0.01^b^ ± 0.01	0.01^ab^ ± 0.00
Taurine	0.30 ± 0.11	0.26 ± 0.12	0.23 ± 0.07	0.26 ± 0.13
**Threonine**	0.07^a^ ± 0.05	0.08^a^ ± 0.04	0.10^b^ ± 0.04	0.11^b^ ± 0.05
Tyrosine	0.02 ± 0.02	0.02 ± 0.01	0.02 ± 0.01	0.01 ± 0.01
Urea	1.62 ± 0.63	1.71 ± 0.89	1.76 ± 0.88	2.01 ± 1.12
**Valine**	0.04^a^ ± 0.04	0.06^b^ ± 0.02	0.06^b^ ± 0.02	0.05^b^ ± 0.01

Significant differences analyzed by one-way ANOVA followed by Tukey’s HSD with a level of significance at p < 0.05. If variables were not normally distributed, statistical significance between time points was analyzed by the Kruskal–Wallis test following the Wilcoxon rank sum test with Benjamini–Hochberg corrected p-values and a level of significance at p < 0.05. Significant differences between time-points are indicated by different letters. Metabolites that significantly differ over lactation are written in bold. Abbreviations: mM; millimolar, SD; standard deviation, *variables normally distributed.

**TABLE 4:** Mean levels (in mM ± standard deviation) of fatty acids and derivatives in human milk from term deliveries sampled weeks 1–2, 3–5, 7–9, and 20–25 postpartum.

*Fatty acids and derivatives*	Week 1-2 (mM ± SD)	Week 3-5 (mM ± SD)	Week 7-9 (mM ± SD)	Week 20-25 (mM ± SD)
Acetate	0.02 ± 0.01	0.04 ± 0.10	0.01 ± 0.01	0.02 ± 0.01
**Butyrate**	0.01^a^ ± 0.02	0.04^b^ ± 0.04	0.08^b^ ± 0.09	0.18^c^ ± 0.17
**Caprate**	0.06^a^ ± 0.06	0.09^ab^ ± 0.08	0.09^ab^ ± 0.06	0.12^b^ ± 0.06
**Caprylate**	0.08^a^ ± 0.08	0.13^ab^ ± 0.11	0.15^bc^ ± 0.12	0.25^c^ ± 0.17
**Choline**	0.14^a^ ± 0.07	0.13^a^ ± 0.09	0.16^a^ ± 0.11	0.23^b^ ± 0.08
**Ethanolamine**	0.08^a^ ± 0.02	0.07^a^ ± 0.02	0.06^b^ ± 0.02	0.06^b^ ± 0.02
**O-phosphocholine**	0.67^a^ ± 0.20	0.60^a^ ± 0.21	0.50^b^ ± 0.18	0.31^c^ ± 0.15
Sn-glycero-3-phosphocholine*	0.49 ± 0.23	0.49 ± 0.23	0.43 ± 0.15	0.49 ± 0.16
**Valerate**	0.02^a^ ± 0.02	0.05^b^ ± 0.05	0.06^b^ ± 0.06	0.15^c^ ± 0.15

Significant differences analyzed by one-way ANOVA followed by Tukey’s HSD with a level of significance at p < 0.05. If variables were not normally distributed, statistical significance between time points was analyzed by the Kruskal–Wallis test following the Wilcoxon rank sum test with Benjamini–Hochberg corrected p-values and a level of significance at p < 0.05. Significant differences between time-points are indicated by different letters. Metabolites that significantly differ over lactation are written in bold. Abbreviations: mM; millimolar, SD; standard deviation, *variables normally distributed.

**TABLE 5:** Mean levels (in mM ± standard deviation) of metabolites related to energy metabolism in human milk from term deliveries sampled weeks 1–2, 3–5, 7–9, and 20–25 postpartum.

*Energy metabolites*	Week 1–2 (mM ± SD)	Week 3–5 (mM ± SD)	Week 7–9 (mM ± SD)	Week 20–25 (mM ± SD)
**2-oxoglutarate**	0.04^a^ ± 0.02	0.05^ab^ ± 0.03	0.05^ab^ ± 0.03	0.07^b^ ± 0.06
Acetate	0.02 ± 0.01	0.04 ± 0.10	0.01 ± 0.01	0.02 ± 0.01
Cis-Aconitate*	0.01^a^ ± 0.00	0.01^b^ ± 0.00	0.01^b^ ± 0.00	0.01^b^ ± 0.00
**Citric acid** *	4.17^a^ ± 1.00	2.98^b^ ± 0.80	2.58^b^ ± 0.71	1.88^c^ ± 0.57
Creatine*	0.06 ± 0.02	0.06 ± 0.02	0.06 ± 0.02	0.06 ± 0.03
**Creatinine** *	0.05^ab^ ± 0.01	0.06^a^ ± 0.01	0.06^a^ ± 0.01	0.04^b^ ± 0.01
**Creatine phosphate**	0.02^a^ ± 0.01	0.02^a^ ± 0.02	0.02^a^ ± 0.02	0.01^b^ ± 0.01
Fumarate	0.00 ± 0.00	0.00 ± 0.00	0.00 ± 0.00	0.00 ± 0.00
Lactate	0.12 ± 0.07	0.13 ± 0.10	0.16 ± 0.33	0.08 ± 0.04
Pyruvate	0.02 ± 0.01	0.01 ± 0.01	0.01 ± 0.01	0.01 ± 0.00
Succinate	0.01 ± 0.01	0.01 ± 0.01	0.01 ± 0.00	0.01 ± 0.00
***Unclassified metabolites***				
2-hydroxybutyrate	0.01 ± 0.00	0.01 ± 0.00	0.01 ± 0.00	0.01 ± 0.01
Hippurate	0.01 ± 0.01	0.02 ± 0.02	0.01 ± 0.01	0.01 ± 0.01
Pantothenate	0.02 ± 0.01	0.02 ± 0.01	0.02 ± 0.01	0.02 ± 0.01

Significant differences analyzed by one-way ANOVA followed by Tukey’s HSD with a level of significance at p < 0.05. If variables were not normally distributed, statistical significance between time points was analyzed by the Kruskal–Wallis test following the Wilcoxon rank sum test with Benjamini–Hochberg corrected p-values and a level of significance at p < 0.05. Significant differences between time points are indicated by different letters. Metabolites that significantly differ over lactation are written in bold. Abbreviations: mM; millimolar, SD; standard deviation, *variables normally distributed.

#### Concentrations of Sugars and Human Milk Oligosaccharides at Distinct Stages of Lactation

The metabolite detected in greatest concentrations at all stages of lactation was lactose, which significantly increased over lactation from 166.72 mM (57.07 g/L) at weeks 1–2 to 200.76 mM (68.72 g/L) in weeks 20–25 (Table 2). Regarding other sugars, glucose exhibited most variation in the first period after birth and significantly increased throughout the first month of lactation, from 0.94 mM in weeks 1–2 to 1.33 mM in weeks 3–5, though remained statistically stable thereafter. Levels of gluconate significantly decreased throughout all lactation stages, from 0.90 mM in weeks 1–2 to 0.25 mM in weeks 20–25 (Table 2).

In general, the HMOs detected in highest levels were 2-FL, 3-FL, and LNT, although they exhibited distinctive changes over the investigated 1–25 weeks of lactation (Table 2). Levels of 2-FL, 3-SL, 6-SL, LDFT, LNDFH I, and LNT were all significantly higher early in lactation. Specifically, 2-FL was significantly more abundant in milk from weeks 1–2 (2.48 mM, 1.21 g/L) compared to all other lactation stages, but levels were not significantly different when comparing between weeks 3–5, 7–9, and 20–25 (Table 2). Levels of 3-SL were significantly more abundant in weeks 1–2 (0.18 mM, 0.12 g/L) compared to all other lactation stages, and between weeks 3–5 (0.147 mM, 0.096 g/L) weeks 7-9 (0.12 mM, 0.08 g/L), the levels significantly decreased. Levels of 6-SL, LNDFH I, and LNT significantly and progressively decreased over the entire investigated period, from 0.95 mM (0.62 g/L 6-SL), 1.19 mM (1.19 g/L, LNDFH I), and 3.82 mM (2.25 g/L, LNT) in weeks 1–2 to 0.08 mM (0.05 g/L, 6-SL), 0.40 mM (0.40 g/L, LNDFH I), and 1.13 mM (0.80 g/L, LNT) in weeks 20–25 (Table 2). In contrast, 3-FL was dominant in later stages of lactation and significantly increased in levels from the first weeks following birth (0.94 mM, 0.46 g/L in weeks 1–2) to later stages of lactation (2.61 mM, 1.27 g/L in weeks 20–25, Table 2).

#### Concentrations of Amino Acids and Derivatives at Distinct Stages of Lactation

Glutamate was detected in the highest concentration out of all free amino acids identified and increased significantly during the first month of lactation from 0.89 (weeks 1–2) to 1.25 mM (week 3.5). Whereas levels of glutamate changed insignificantly after 1 month of lactation, levels of glutamine increased progressively and significantly at all time points from 0.07 mM in weeks 1–2 to 0.59 mM in weeks 20–25 (Table 3). Similarly, levels of O-acetylcarnitine significantly decreased throughout all lactation stages from 0.04 mM in weeks 1–2 to 0.01 mM in weeks 20–25 (Table 3).

As in the case of glutamate, other amino acids likewise changed significantly throughout the first month of lactation only. Alanine and valine levels significantly increased during the first month of lactation from 0.18 mM (alanine) and 0.04 mM (valine) at weeks 1–2 to 0.25 mM (alanine) and 0.06 mM (valine) at weeks 3–5. In contrast, lysine decreased the first month of lactation from 0.04 mM to 0.02 mM and likewise remained stable from weeks 3–5 and onward (Table 3). Finally, levels of other amino acids were constant throughout the first month of lactation and only changed significantly after weeks 3–5. This included levels of threonine, leucine, and 2-aminobutyrate. Levels of threonine were constant throughout the first month of lactation and increased in levels from weeks 1–2 and 3–5 to 7.9 week (0.10 mM). Likewise, levels of leucine were significantly increased when comparing weeks 1–2 (0.02 mM) and 7–9 (0.03 mM) or 20–25 week (0.03 mM). Levels of 2-aminobutyrate were constant at the first month of lactation from weeks 1–2 to 3–5 at a level of 0.010 mM and thereafter increased progressively and significantly to 0.021 mM in weeks 20–25, whereas levels of betaine were significantly increased when comparing weeks 1–2 (0.06 mM) and 20–25 (0.08 mM) only.

#### Concentrations of Fatty Acids and Derivatives at Distinct Stages of Lactation

Regarding the fatty acids, butyrate (0.18 mM), caprate (0.12 mM), caprylate (0.25 mM), and valerate (0.15 mM) were detected in highest levels in late lactation at weeks 20–25 (Table 4). When comparing weeks 1–2 and 7–9 and/or 20–25, levels of caprate and caprylate were significantly increased, and levels of butyrate and valerate increased during the first month of lactation (weeks 1–2 to 3–5) and then remained stable before significantly increasing in weeks 20–25.

Moreover, O-phosphocholine levels were unchanged during the first month of lactation (0.67–0.60 mM in weeks 1–2 to 3–5), but then decreased significantly at lactation stage week 7–9 (0.50 mM) and weeks 20–25 (0.31 mM, Table 4). Similarly, levels of ethanolamine did not significantly vary throughout the first month of lactation (weeks 1–2 vs. 3–5) but decreased significantly at weeks 7–9 (0.06 mM), after which the levels again were statistically stable (0.06 mM at weeks 20–25, Table 4). Levels of choline were statistically constant until weeks 7–9 (0.16 mM), after which the levels significantly increased to weeks 20–25 of lactation (0.23 mM).

#### Concentrations of Energy Metabolites and Unclassified Metabolites at Distinct Stages of Lactation

We observed that citric acid, pyruvate, cis-aconitate, fumarate, and succinate all tended to cluster on the left side of the first PC in the PCA model and thus correlated with samples from earlier stages of lactation (Figures 1A,B). Based on univariate data analysis, citric acid levels significantly decreased over lactation from 4.17 mM in weeks 1–2 to 1.88 mM in weeks 20–25 (Table 5). Cis-aconitate was detected in highest level at weeks 1–2 (0.01 mM) and decreased significantly in the first month of lactation, weeks 3–5, after which the levels did not significantly change from weeks 3–5 to 20–25 (Table 5). Neither pyruvate, fumarate nor succinate changed significantly throughout lactation, whereas 2-oxoglutarate increased toward the late lactation from 0.04 mM in weeks 1–2 to 0.07 mM in weeks 20–25 (Table 5). Finally, levels of creatinine were significantly increased at weeks 3–5 and 7–9 (0.56 mM at both time points) compared to weeks 20–25 (0.40 mM), whereas levels of creatinine at weeks 1–2 were not significantly different from the remaining lactation stages (Table 5).

## Discussion

Human milk is nutritionally both a highly complex and complete food provided for infants to ensure optimal development early in life, and besides from macronutrients consists of bioactive compounds and metabolites deriving from maternal metabolism. The maturation of analytical technology in metabolomics is still ongoing (31). However, data acquired using different technologies should be comparable if absolute quantitation of metabolites is performed. NMR has previously been a widely used technique in milk-based metabolomic studies, as NMR offers absolute quantitation and measures the most abundant metabolites in the μm range and up (16, 17). However, care must be taken when comparing across studies as several parameters might influence the milk metabolome, where sample collection is most prominent (13). The most dominant sampling technique is spot sampling, where a small amount of fore or hind milk is collected. Less common is complete emptying of the breast, as this might interfere with the mother’s natural breastfeeding of the baby. The composition of HM is known to vary over lactation moving from colostrum to mature milk (around 1 month after birth), though less is known about the composition of HM metabolome from clinically healthy mothers delivering at term throughout lactation, beyond 1 month postpartum. Overall, we find that the metabolome of HM continuously varies throughout lactation and some changes are distinctive for milk excreted after 1 to 2 months of lactation. The trend for lactose, medium- and short-chain fatty acids, such as butyrate, caprate, and caprylate, the amino acids glutamate and glutamine to positively correlate with progressing lactation, and citric acid and HMOs to negatively correlate with progressing lactation is in accordance with similar tendencies observed by others (15–17, 23, 32). Univariate data analysis largely substantiated the tendencies observed in the multivariate data analysis. Levels of gluconate, citric acid, cis-aconitate, ethanolamine, and O-acetylcarnitine were significantly higher early in lactation than in later stages. In contrast, levels of lactose, glucose, glutamate, valine, valerate, threonine, alanine, glutamine, butyrate, 2-aminobutyrate, caprate, and caprylate were found to significantly increase from weeks 1–2 to later stages of lactation.

### Sugars and Human Milk Oligosaccharides

Compared to the works of others, lactose has been observed to significantly increase over lactation in HM from term deliveries over 1 month of lactation (16) and in HM sampled up to 3 months postpartum (15). The study reported by Spevacek et al. (16) used milk collected by pumping a full breast, whereas Andreas et al. (15) predominantly collected hind milk, and fore milk is collected in this study. Despite the differences in collection strategy, there is an agreement of the results and also confirmed here. Glucose changed in the levels in accordance with previous reports (16), although it remained stable after 1 month of lactation. In another study, significant increases in levels of glucose were only observed throughout the first 10 days postpartum, but not at later stages of lactation (6–10 vs. + 10 days, latter spanning up to 3 months postpartum) (15). Gluconate is an oxidized form of glucose and, to the best of our knowledge, no other metabolomic studies have to date made similar observations. One study observed a positive correlation between gluconate and week 4 of lactation (*n* = 9) compared to week 1 (*n* = 10) (33), and another reported no correlation with lactation stage (17).

Human milk oligosaccharides particularly varied throughout lactation, as observed both from the multivariate data analysis and the univariate data analysis. Human milk oligosaccharides are acknowledged prebiotic components of HM (34), with high structural diversity (21), and their concentrations have been reported to be dependent on maternal phenotype (20, 35, 36), term- or preterm delivery (17), maternal diet (37), and lactation stage (38). Maternal phenotype specifically relates to secretor status and Lewis gene type of the mother, which determine the chemical composition of fucosylated HMOs in the milk. In line with other findings, 19% of mothers were classified as non-secretors (16, 39), and of the secretor mothers, six were identified as Lewis negative, corresponding to 10% of the total cohort, and none were non-secretors and Lewis negative, consistent with the expectation for this phenotype to be present in 1% of the population (39). We found that secretor status mainly discriminated observations along the fifth PC in a multivariate data analysis, thus only contributing to the variation and composition of HM metabolites in minor degree. Generally, other studies have observed a greater contribution of secretor status on the metabolome of HM (16, 17). However, we observed clear trends in terms of changes in levels of HMOs over lactation. Levels of all HMOs except 3-FL were significantly higher in early stages of lactation. This trend is in line with previous results (38). Multiple interactions between HMOs and the infant gut microbiota and environment have been extensively reviewed previously (6, 34, 40). Research has suggested that 2-FL and 3-FL are negatively correlated (38), and studies have demonstrated that both 2-FL and 3-FL promote the growth of infant gut symbionts, enhance production of short chain fatty acids, and improve intestinal mobility and epithelial barrier function (6). Thus, despite overall decreasing levels of HMOs identified in this study over lactation and the lack of α1-2 fucosylated HMOs in HM from non-secretor mothers, increasing levels of 3-FL over lactation provide continuous bioactivity to the neonatal gut environment. In addition to variation dependent on lactation stage, we observed a tendency for maternal secretor status to affect the concentration of 3-FL, LNT, and 3-SL in HM, whereby non-secretor mothers tended to have higher concentrations of these HMOs in their milk. Although we did not conduct statistical analyses on these observations owing to small sample size of non-secretor mothers, the tendency for 3-FL and LNT to be increased in HM from non-secretor mothers delivering birth at term has been observed elsewhere (36, 41).

### Amino Acids

Glutamine and glutamate are generally present in highest concentration out of all free amino acids in HM (42), and both glutamine and glutamate have previously been observed in metabolomic studies to significantly increase over or positively correlate with lactation (15–17). However, contrary to the results presented in this study, Andreas et al. (15) found glutamine levels to significantly increase between the first 5 days of lactation and + 10 days of lactation, but not when comparing lactation day 6–10 with + 10 days using a multi-omics approach (*n* = 57). The results presented in the study of Spevacek et al. (16) conducting ^1^H NMR metabolomics on HM from a cohort of term deliveries (*n* = 15) collected at days 0–5 (colostrum), 14 (transitional HM), and 28 (mature HM) indicate that both glutamine and glutamate significantly increase over the first month of lactation, consistent with the present findings. Glutamine and glutamate are important for growth of the neonate’s gut as enterocytes of the neonate utilize these amino acids for metabolism leading to improved barrier function. As such, glutamine is utilized in the intestine and converted to alanine, during which NADH and FADH_2_ are generated, providing energy for gut enterocytes (18). Adding to this, both amino acids interact with the immune system and gut microbiota (43). Moreover, glutamine is widely involved in various metabolic pathways in the human body, including the regulation of the immune system and cell functions (44). The increased concentration of these amino acids over lactation could facilitate gut maturation and growth of the infant by providing proteinogenic amino acids readily involved in protein synthesis (43, 44). In support of this, a study observed significantly increased glutamate levels and increased difference in glutamine levels from 1 to 4 months in HM for infants with faster weight gain in the first 4 months postpartum (45). Other amino acids significantly changed in levels over lactation, of which some significantly changed the first month of lactation only (alanine, valine, methionine, and lysine), whereas others were constant at the first month of lactation and changed significantly with progressing lactation afterward (threonine, leucine, 2-aminobutyrate). Alanine, valine, lysine, and methionine showed similar trends in the present univariate data analysis as described by others (15, 16, 46), though, in the multivariate data analysis, valine was not strongly correlated with later stages of lactation. Moreover, whereas the levels of threonine were constant throughout the first month of lactation, in line with previous observations (16), we find that after weeks 3–5, levels of threonine increased significantly, which is not in line with previous observations (46).

Valine, methionine, lysine, leucine, threonine, and phenylalanine are all indispensable amino acids for the new-born baby (46). In the mammary gland, a proportion of branched-chain amino acids (BCAAs) is catabolized for synthesis of glutamate and glutamine (47), which increased over lactation. The proportion of BCAAs excreted in milk is important for infant growth and development early in life, as they provide nitrogen for protein synthesis, neurotransmitter synthesis and interact with the immune system (48, 49). In support of this, a study observed that BCAAs and insulin-trophic amino acids were associated with preterm infants experiencing fast growth at the first month postpartum (50), thereby underlining the potential importance of particularly BCAAs for infant growth early in lactation.

We found higher levels of 2-aminobutyrate after 7–9 weeks of lactation, although another study observed significantly higher levels of 2-aminobutyrate the first month of lactation in HM from term deliveries (16), and abundance of 2-aminobutyrate was significantly increased throughout the first 6 months of lactation compared to after 6 months of lactation (*n* = 130) in a third study (51). Although not fully in agreement with this study, the aforementioned results, combined with the ones in the present study, demonstrate that, throughout lactation and between lactation stages, 2-aminobutyrate is among the metabolites continuously changing in concentration, potentially reaching a maximum around the first 6 months of lactation. 2-aminobutyrate is a non-proteinogenic amino acid involved in catabolism of methionine, threonine, and serine. Associations have been observed between lower levels of 2-aminobutyrate in HM and inflammatory bowel disease for mothers (52), and higher levels in HM have been reported for overweight/obese mothers at 1 month of lactation (26).

Additionally, levels of O-acetylcarnitine changed significantly throughout all the stages of lactation. This trend is in accordance with the results of others (16). The implications of O-acetylcarnitine (acetyl-L-carnitine) in HM are not well characterized. In the cells of the human body, O-acetylcarnitine is formed from the transfer of an acetyl-group to carnitine (53). Both carnitine and O-acetylcarnitine are involved in fatty acid oxidation, by which the carnitine shuttle facilitates the transport of acyl-CoA derivatives of long-chain fatty acids across the mitochondrial membrane for oxidation (54). Various species of acylcarnitines have been reported to be implicated in insulin sensitivity, with long chain acylcarnitines specifically being indicative of development of type 2 diabetes, and increased levels of acylcarnitines from BCAA metabolism being reported in HM from overweight/obese mothers (26, 54, 55). However, acetylcarnitine has been associated with improvement of glucose tolerance, metabolic flexibility, and neurological function (53, 56, 57). How levels of O-acetylcarnitine in HM might affect infant metabolism is unresolved although, as an acetylated form of carnitine, O-acetylcarnitine is readily absorbed in the gut (53), with the potential of assisting uptake of carnitine in the infants. Despite no observed significant change in levels of carnitine over lactation in this study, changing levels of O-acetylcarnitine over lactation could reflect shifts in metabolic activity of the mammary gland involving glucose metabolism and fatty acid oxidation.

Compared to the works of others, betaine, leucine, and phenylalanine have been found to differ from that presented in this study (16, 17, 46). Levels of betaine were significantly increased when comparing weeks 1–2 and 20–25 only, whereas others have found decreased levels of betaine over lactation (16, 17). Research to date suggests that multiple factors influence the composition of free amino acids in HM besides the lactation stage. As such, variation in amino acid content of HM has been observed to depend on the infant sex (45) and maternal BMI (28). Moreover, variations in amino acid composition within a single feed have been observed, with increased levels of free amino acids, phenylalanine, threonine, alanine, valine, glutamine, and serine in foremilk (58).

### Fatty Acids and Derivatives

The fatty acids detected in this study using ^1^H NMR spectroscopy – butyrate, caprate, caprylate, and valerate all significantly increased over lactation and were found to be positively correlated with samples of late lactation in a multivariate data analysis. Meng et al. investigated macronutrient levels and fatty acid profiles in HM from same cohort as this study and found a tendency for both caprate and caprylate to increase over 24 weeks of lactation (5.51 to 6.21 mg g^–1^ and 1.83 to 2.06 mg g^–1^ respectively), though the results were not significant. However, a significant impact of maternal BMI on levels of saturated fatty acids, including caprylate, and an additional effect of infant gender on levels of caprylate in HM at 24 weeks of lactation were identified^2^. In another cohort studying HM from mothers delivering term (*n* = 30), spanning from colostrum to mature milk (comprising milk sampled once at 5–33 weeks of lactation) butyrate, caprate, and caprylate correlated positively with mature milk (17), in line with the present results. Increased levels of short- and medium-chain fatty acids over lactation have been suggested to be related to mammary gland maturation and coherently upregulated *de novo* fatty synthesis (59). A recent review highlighted how medium-chain fatty acids affect immune system processes and gut microbiota (60) over and above serving as an energy source. Concentrations of butyrate, caprate, and caprylate have been reported to increase over lactation in other studies (8, 17, 61). Furthermore, the influence of maternal diet has been investigated in an intervention study (*n* = 14), which indicated that a low-fat diet results in higher concentrations of medium-chain fatty acids (62).

Levels of valerate followed the same pattern over lactation as described for butyrate, caprate, and caprylate, although no other studies investigating HM metabolome have reported on valerate. The origin or importance of valerate in HM is not understood, but others highlight valerate produced by the gut microbiota to induce growth of intestinal epithelium and protection against diseases, such as colitis and cardio-metabolic diseases (63). It is possible that valerate detected in our samples derives from microbial metabolism by species of the HM microbiome (64), which change significantly from birth to 24 weeks of age (65).

In agreement with the present results, others found the levels of O-phosphocholine to be constant throughout the first month of lactation (16) and decrease significantly after 1-month of lactation in a pilot study using a single donor (32). In contrast, results from another cohort sampling HM at 3 days and 6 months postpartum (*n* = 31) observed the opposite trend for O-phosphocholine (23) and increasing levels of ethanolamine over 1 month of lactation and decreasing levels of choline have been observed (16). Large inter-individual variability in HM content has been reported for levels of ethanolamine and O-phosphocholine dependent on regional differences (25), and in choline content of HM between mothers, as free choline in HM was found to be correlated with levels of free choline, phospholipid-bound choline and glycerophosphocholine in the maternal serum (19). O-phosphocholine, choline, and ethanolamine in HM derive from the milk fat globule membrane, as these are common head groups for phospholipids, but also found as free components in the milk (66, 67). While ethanolamine is synthesized into phosphatidylethanolamine, O-phosphocholine and choline are intermediates in the synthesis of phosphatidylcholine (66, 68).

### Metabolites Related to Energy Metabolism

Of metabolites related to energy metabolism, we observed that citric acid, pyruvate, cis-aconitate, fumarate, and succinate all tended to cluster on the left side of the first PC in the multivariate data analysis and thus correlated with samples from earlier stages of lactation. Of these, the univariate data analysis reflected significant variation in levels of citric acid, cis-aconitate, and 2-oxoglutarate over lactation. As mentioned previously, other studies have identified increased levels of citric acid in earlier stages of lactation compared to later stages of lactation (15, 16, 32, 51), though some discrepancies exist in terms of variation in metabolites related to energy metabolism found in HM. In a study conducting HM metabolomics across various geographical regions with HM sampled at 1 month of lactation (*n* = 109), 2-oxoglutarate, citric acid, and lactose were positively correlated, although with region-specific variation in levels of citric acid and 2-oxoglutarate (22). In the study by Spevacek et al. (16), levels of 2-oxoglutarate decreased throughout the first month of lactation, opposite to what was observed in this study. Moreover, fumarate and pyruvate levels significantly decreased over 1 month of lactation (16), whereas neither levels of cis-aconitate nor citric acid significantly changed, in contrast to the present results (Table 5), and results of others (15, 17). Citric acid, cis-aconitate, fumarate, succinate, pyruvate, and 2-oxoglutarate are all associated with tricarboxylic acid (TCA) cycle (69), and correlations could reflect the metabolic activity of the mammary gland. In a study comparing the metabolome of the tissue of mammary gland, milk, rumen fluid, and urine from lactating and non-lactating cows, TCA cycle was particularly upregulated in mammary gland of lactating cows based on pathway analysis, with citric acid identified as a biomarker of lactation and expressed in greater levels (70). 2-oxoglutarate is a metabolite involved in both carbon metabolism and nitrogen metabolism, serving as an intermediate of TCA cycle and an intermediate in nitrogen-assimilatory reactions, respectively (71). Thus, the increased levels of 2-oxoglutarate observed in this study might likewise partly be linked to glutamate synthesis, as both glutamine and glutamate were observed to be increased in later stages of lactation. Overall, higher levels of citric acid and cis-aconitate early in lactation, lower levels of 2-oxoglutarate, and increased levels of glutamine and glutamate with progressing lactation were observed. As partly implied by Smilowitz et al. (36), part of these correlations could be explained by the hypothesis that, later in lactation, citric acid and potentially cis-aconitate are partly being utilized for 2-oxoglutarate production in the mammary gland and eventually glutamate synthesis, thus providing increased nitrogen load for the infant for amino acid synthesis.

### Discrepancies Between Analyses and Limitations of Study

We found some discrepancies in the tendencies reflected by the multivariate data analysis compared to the univariate data analysis. Specifically, levels of 2-oxoglutarate, choline, and O-phosphocholine changed significantly over lactation in the univariate analysis, but this tendency was not evident in the PCA model, as the metabolites have a low impact on the first PC. On the other hand, pyruvate and fucose seemed to correlate with samples from earlier stages of lactation in the PCA model and urea with later stages of lactation, but the levels were not significantly different in univariate data analysis. Finally, creatine phosphate decreased significantly over lactation when comparing weeks 20–25 with the remaining lactation stages but, in the multivariate data analysis, creatine phosphate correlated with samples from later stages of lactation on the first PC. However, this correlation could possibly reflect correlation with samples from weeks 3–5 to 7–9 as these largely overlapped in the PCA. Part of these discrepancies between the multivariate and univariate analysis may be ascribed to the fact that the PCA model comprised 57.4% of the variation in the dataset, and lactation stage explained about 33% of the variation in the data, leaving some residual variation not accounted for in the multivariate analysis. Furthermore, PCA models also take co-variance into consideration, unlike univariate analyses which do not. Moreover, in the multivariate data analysis, some observations strongly correlated with free amino acids including tyrosine, isoleucine, leucine, and lysine. These did not derive from the same donor nor consistently the same time point, and the reason for the similarities of these samples in terms of levels of lysine, leucine, isoleucine, and tyrosine cannot be explained with the information available.

A strength of this study is the inclusion of multiple time points postpartum, and the duration of the study moving beyond 1 month of lactation to 6 months of lactation. This enables analysis of changes in HM beyond the state of maturity and at various lengths of lactation. Previous studies have investigated variation over lactation in HM from term mothers longitudinally (1, 16, 23, 26, 27, 52, 72, 73), although the research focus has included mothers delivering term with pathological conditions (23, 52, 72), inclusion of samples from various lactation stages ranging from 1 week postpartum to 6 months, and with maximum three HM collections throughout the study period. For example, one study collected HM at 3 days and 6 months postpartum (23), one collected after 1 month and then again at 6 months (26), three studies sampled three times throughout the first month after birth (16, 72, 73), one after 3 and 6 months (52), and one study collected the first milk (colostrum), mature milk (2 months postpartum), and milk at 6 months (27). Thereby, this study provides insights into the HM metabolome of mature milk, and we demonstrate that even mature milk progressively varies over lactation with specific changes in HM metabolome not observed in HM from 1 month of lactation. Limitations of this study are the limited ethnicity and geographical region of included participants. The cohort is based on Irish women, and so the results presented could be influenced by geographical differences (25). Moreover, several participants did not deliver milk samples at all time points, by which these observations were single-point observations rather than longitudinal, and we have not collected samples between 9 weeks of lactation and 20 weeks of lactation, leaving a gap in terms of understanding the development of HM metabolome within this timeframe. Evidently, our results are restricted by the limitations of our chemical analysis, as ^1^H NMR-based metabolomics has a high lower limit of detection in the μm range than MS-based methods (nM range) and mainly detects constituents in the aqueous phase, such as amino acids, saccharides, and energy metabolites, but cannot detect the full range of lipid species found in HM (15, 74). Nevertheless, the results of this study add to the knowledge of the development of the HM metabolome from term deliveries in the first period after birth from 1 week to about 6 months postpartum. Moreover, we comprehensively characterize HM composition from macronutrient level to metabolite level, including fatty acid profiles in HM of mothers delivering term over 6 months of lactation. Furthermore, we have also recently reported on the HM microbiome in this cohort of lactating women over the course of lactation from birth to 6 months (75).

Though some variations in metabolite levels observed in this study have been observed in previous studies as discussed, we here add to the knowledge on HM metabolome as some changes in metabolite levels observed in this study are predominantly found in mature milk after 1 to 3 months of lactation. However, whereas for other metabolites, we demonstrate that although it has been previously observed in several studies that particularly lactose, glutamate, alanine, and 2′FL change in levels over lactation, we here observe that the changes are significant within the first month of lactation from transitional to mature milk, after which the levels are stable. As previously mentioned, glutamate and glutamine have previously been observed to increase in levels over 1 month of lactation in another metabolomic study, though we here show that glutamine continuously increase in levels in late mature milk, whereas levels of glutamate remain stable after 1 month of lactation. Increased levels of glutamine and glutamate over lactation may provide nitrogen for protein synthesis and gut maturation coherently with increased nutritional demand of the infant. Moreover, TCA cycle intermediates, including citric acid, have been observed to correlate with early lactation, and studies have observed distinct tendencies in the variation in levels over lactation, with this study adding to the knowledge on metabolites related to energy metabolism. Increased levels of TCA cycle intermediates early in lactation could reflect a shift in energy metabolism in the mammary gland throughout lactation, potentially to accommodate a changing need in the infant or be related to mother’s milk production. Increasing levels of butyrate, caprate, caprylate, and valerate and variation in levels of fatty acid derivatives, including choline, O-phosphocholine, and ethanolamine, could reflect shifts maternal metabolism or related to the composition of the milk fat globule membrane. Moreover, whereas the concentration of most HMOs declines over lactation, that of 3-FL increases continuously, offering a prebiotic component to the gut environment of the infant. Combined, these results indicate that the composition of HM metabolome continuously changes throughout lactation to potentially accommodate the growing infant’s needs and/or reflect shifts in mammary gland metabolism. However, the biological implications of these variations are not investigated in this study, and interpretations of the relations are therefore purely speculative. Nevertheless, these changes influence both nutritional and non-nutritional components provided for the infant. Thus, the results of this study support further in-depth research into the dynamic nature of late mature HM to investigate the impact on infant growth and development.

## Conclusion

This study demonstrates how HM from mothers delivering at term changes over a period beyond the state of maturity from early lactation to late lactation (weeks 1–25 postpartum). Besides from the variation in agreement with similar studies in terms of levels of lactose, glutamine, glutamate, butyrate, caprate, caprylate, citric acid, and HMOs, we additionally found O-acetylcarnitine, gluconate, ethanolamine, and cis-aconitate to be correlated positively with early lactation, and 2-aminobutyrate, and valerate to be correlated positively with later lactation. Some metabolites change in concentration mainly throughout the first month after birth and afterward remain stable whereas, for others, the concentration is dynamic and continuously changes throughout lactation. This dynamic change in concentration is particularly evident for 6-SL, gluconate, glutamine, LNT, LNDFH I, and O-acetylcarnitine.

## Data Availability Statement

The raw data supporting the conclusions of this article will be made available by the authors, without undue reservation.

## Ethics Statement

The studies involving human participants were reviewed and approved by Clinical Research Ethics Committee of Cork Teaching Hospitals, Cork, Ireland. The patients/participants provided their written informed consent to participate in this study.

## Author Contributions

ALK, EL, CS, and CAR: conceptualization. EL and UKS: methodology. KOP: formal analysis, investigation, writing—original draft preparation, and visualization. ALK and UKS: resources, project administration, and funding acquisition. CAR, ALK, and UKS: data curation. ALK, FM, UKS, and JFY: writing—review and editing. UKS and JFY: supervision. All authors have read and agreed to the published version of the manuscript.

## Conflict of Interest

The authors declare that the research was conducted in the absence of any commercial or financial relationships that could be construed as a potential conflict of interest.

## Publisher’s Note

All claims expressed in this article are solely those of the authors and do not necessarily represent those of their affiliated organizations, or those of the publisher, the editors and the reviewers. Any product that may be evaluated in this article, or claim that may be made by its manufacturer, is not guaranteed or endorsed by the publisher.

## Footnotes

1
http://cran.r-project.org

2 Meng F, Uniacke-Lowe T, Lanfranchi E, Meehan G, O-Shea CA, Dennehy T, et al. *A Longitudinal Study of Macronutrients Levels, Plasmin Activity, and Fatty Acid Profile in Human Milk*. (personal communication, manuscript in preparation).
