# Supplementary material for: Dynamic Changes in the Human Milk Metabolome Over 25 Weeks of Lactation
Source: Front Nutr. 2022 Jul 14;9:917659. doi: 10.3389/fnut.2022.917659 (PMC9331903; doi:10.3389/fnut.2022.917659)
Supplement: Supplementary file 1 [file Data_Sheet_1.PDF]

Supplementary Table 1: Levels of human milk oligosaccharides at different time-points in human milk dependent on secretor status of mother. Levels of human milk oligosaccharides identified and quantified in human milk from mothers delivering at term (n = 100) collected at four different time-points from week one to week 25 of lactation. Mean levels are indicated in mM ± standard deviation[1]. Levels of human milk oligosaccharides are divided according to both lactation stage and secretor status of mother. Number of observations *n* at each lactation stage from each secretor group is indicated in round brackets. **Abbreviations**: **FL**; fucosyllactose, **LDFT**; lactodifucotetraose, **LNDFH**; lacto-N-difucohexaose, **LNT**; lacto-N-tetraose, **NA**; not available, **NS**; non-secretor, **S**; secretor, **SL**; siallylactose

HMOs (mM)	Week 1-2<br>S (n = 22)<br>NS (n = 5)	Week 3-5<br>S (n = 23)<br>NS (n = 5)	Week 7-9<br>S (n = 25)<br>NS (n = 6)	Week 20-25<br>S (n =14)<br>NS (n = 1)
**2FL**				
S	2.481 ±0.737	1.826 ±0.759	1.755 ±0.620	1.310 ±0.418
NS	NA	NA	NA	NA
**3FL**				
S	0.615 ±0.379	1.237 ±0.636	1.365 ±0.843	2.430 ±0.903
NS	2.360 ±0.695	3.294 ±0.734	5.435 ±1.145	5.081 ±NA
**3SL**				
S	0.173 ±0.048	0.143 ±0.035	0.115 ±0.044	0.119 ±0.023
NS	0.211 ±0.059	0.187 ±0.075	0.126 ±0.007	0.194 ±NA
**6SL**				
S	0.964 ±0.242	0.560 ±0.148	0.308 ±0.133	0.078 ±0.041
NS	0.874 ±0.246	0.564 ±0.168	0.402 ±0.118	0.052 ±NA
**LDFT**				
S	0.681 ±0.737	0.507 ±0.590	0.409 ±0.418	0.377 ±0.137
NS	NA	NA	NA	NA
**LNDFH I**				
S	1.194 ±0.430	0.952 ±0.325	0.700 ±0.294	0.404 ±0.185
NS	NA	NA	NA	NA
**LNT**				
S	3.398 ±0.840	2.542 ±0.829	1.767 ±0.673	1.087 ±0.522
NS	5.665 ±0.978	4.546 ±1.697	2.758 ±0.863	1.674 ±NA

[1] Statistical analyses on differences between secretor and non-secretors were not conducted owing to small sample size of non-secretors.

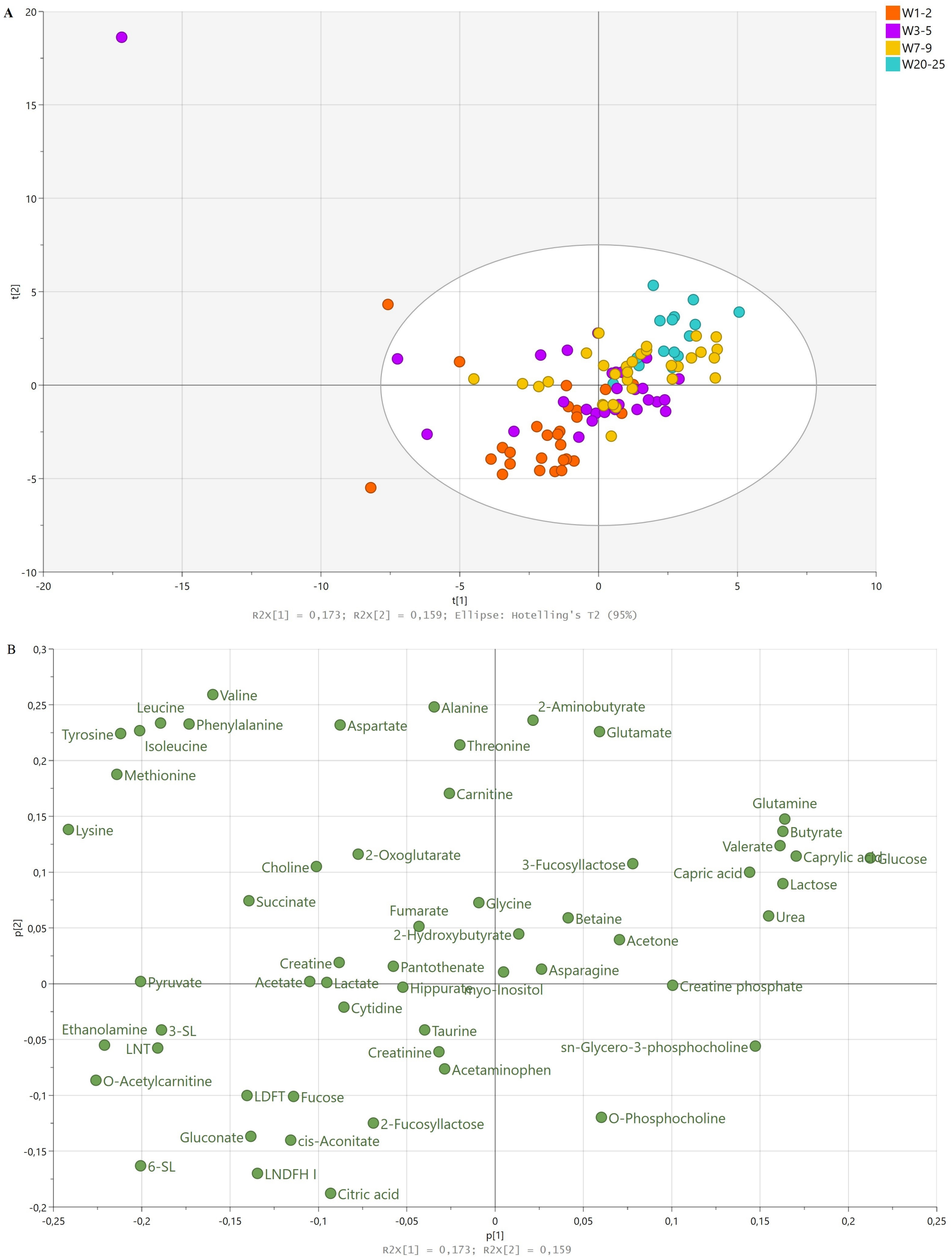

Supplementary Figure 1: **Principal component analysis with deviating sample.** Principal component analysis of human milk samples collected at week one to week 25 after birth (n = 101). **A**: Scores scatter plot of 101 human milk samples with outlier visual in upper left corner. Color-coded according to time-point collected with key to the right. **B**: Corresponding loadings scatter plot. **Abbreviations**: **3-SL**; 3-sialyllactose, **6-SL**; 6-sialyllactose, **LDFT**; lactodifucotetraose, **LNDFH**; lacto-N-difucohexaose, **LNT**; lacto-N-tetraose, **W**; week

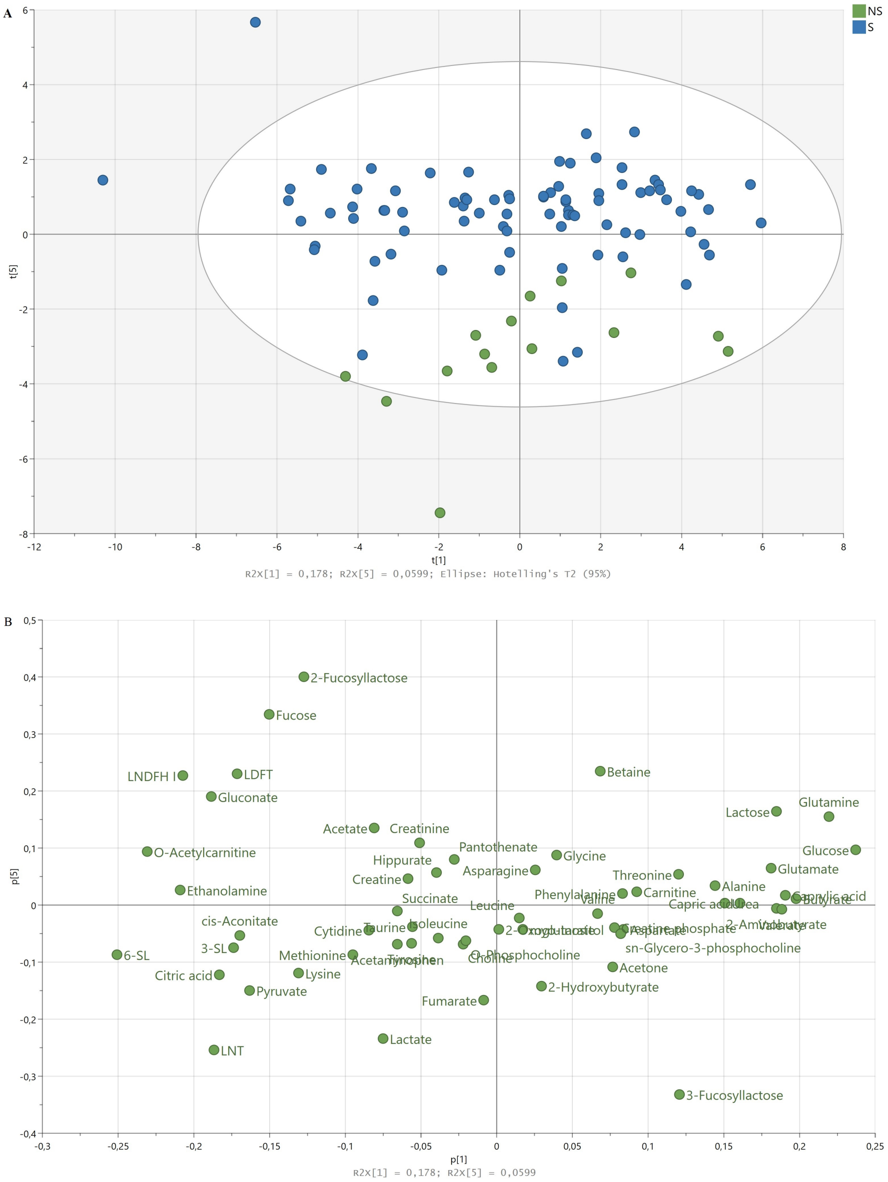

Supplementary Figure 2: Principal component analysis depicting principal component 1 and 5, samples colored according to secretor status of mother. Principal component analysis of human milk samples from mothers delivering at term (n = 100), sampled from week one to week 25 postpartum. Principal component 1 and 5 depicted with the later separating samples according to secretor status. **A**: Scores scatter plot with samples colored according to secretor status and key to right. **B**: Corresponding loadings scatter plot. **Abbreviations**: **3-SL**; 3-sialyllactose, **6-SL**; 6-sialyllactose, **LDFT**; lactodifucotetraose, **LNDFH**; lacto-N-difucohexaose, **LNT**; lacto-N-tetraose, **NS**; non-secretor, **S**; secretor
